# Development of a rapid plasma decontamination system for decontamination and reuse of filtering facepiece respirators

**DOI:** 10.1063/5.0067730

**Published:** 2021-10-07

**Authors:** Minkwan Kim, John Lawson, Rodolphe Hervé, Henrike Jakob, Bharathram Ganapathisubramani, Charles W. Keevil

**Affiliations:** 1 Aeronautics and Astronautics Engineering Department, University of Southampton, Southampton SO16 7QF, United Kingdom; 2 School of Biological Sciences, University of Southampton, Southampton SO17 1BJ, United Kingdom

## Abstract

The COVID-19 pandemic has caused a high demand for filtering facepiece respirators (FFRs), which has brought global challenges in sustaining the supply chain for FFRs. Because respirators are basic personal protective equipment to protect frontline healthcare workers against COVID-19, the chronic, global shortage of N95/N99 masks is one of the most urgent threats to our collective ability to save lives from the coronavirus. The reuse of masks may need to be considered as a crisis capacity strategy to ensure continued availability even though most of the masks are considered one-time use. Moreover, environmentalists warn that single-use masks add to the glut of plastic pollution, threatening the health of oceans and marine life. In this study, we develop a method to decontaminate respirators to reuse filtering facepiece respirators. Samples of SARS-CoV-2 are applied to the 4 × 4 cm^2^ samples of FFP2 and FFP3 respirator materials. The filtration efficiency of plasma treated samples is measured using a planar particle image velocimetry technique with a neutrally charged polydisperse aerosol particle of NaCl. The measured viral decontamination and filtration efficiencies show that the developed plasma decontamination system can achieve a 4-log reduction for the coronavirus without reducing the filtration efficiency of masks after 5-min plasma exposure. The developed plasma decontamination system demonstrates the feasibility to tackle the acute shortages of FFRs in many countries and their environmental and economic burdens against discarding reusable masks.

## I. INTRODUCTION

Filtering facepiece respirators (FFRs) are a piece of basic personal protective equipment (PPE) to protect frontline healthcare workers and the public against COVID-19. Class P2 (FFP2) and class P3 (FFP3) FFRs have the ability to filter out 94% and 99% of particles at 0.3 *μ*m in size.^1^ In the UK alone, more than 2M FFRs are required for healthcare workers, including paramedics, doctors, and nurses who are on the front line of the fight against COVID-19.^2^ Globally, the World Health Organization (WHO) and many countries, including the US and the EU, recommend or mandate wearing masks in public areas,^3,4^ which is significantly increasing the demand for FFRs. A recent UK Foreign, Commonwealth & Development Office (FCDO) report has predicted that the demand for masks will continue to increase through 2021 even if the rollout of vaccination campaigns is considered.^5^ An acute shortage of FFRs means that health workers and first responders must try to stem the pandemic without adequate protective gear.

In addition, environmentalists warn that the rise in disposable FFRs being used to prevent the spread of coronavirus could add to the glut of plastic pollution, threatening the health of oceans and marine life. A recent study has estimated that a new N95 respirator per patient encounter might require 7.41 × 10^9^ respirators, cost $6.38 × 10^9^, and generate ∼84 000 tons of waste in the USA over 6 months.^6^ Although disposable FFRs are made for single-use, they can be reused for a limited time if there is no risk of contamination through the deposition of infectious particles on the surface. Safe decontamination methods, therefore, can reduce the acute shortages of masks and their environmental and economic burdens.

Currently, several activities are being performed to find methods for mask decontamination as a contingency capacity strategy to conserve available supplies for healthcare environments during a pandemic, which include ultraviolet germicidal irradiation (UVGI),^7^ vaporous hydrogen peroxide (VHP),^8^ and moist heat.^9^ Although these methods can effectively inactivate SARS-CoV-2 and other pathogens on FFRs,^2,10,11^ the performance of the decontaminated FFR can be negatively impacted. For example, electrostatic filters and hydrophobic coatings on a mask can be damaged by decontamination agents, such as benzalkonium chloride, ethanol (70% or higher), and chlorhexidine.^11,12^ Although decontamination with bleach can reduce degradation in the filtration performance of a mask, it leaves chemical residues.^13^ Therefore, FFRs decontaminated using these methods are not suitable for reuse.

Non-thermal plasma, also called cold plasma, can be an alternative for the safe decontamination process of FFRs. Previous studies showed that non-thermal plasma can rapidly inactivate 99.9% of various viruses.^13,14^ Although non-thermal plasma is not listed in the Environmental Protection Agency (EPA) N-list as a disinfectant against SARS-CoV-2, hydrogen peroxide is listed in this list, which inactivates microbes by producing highly reactive hydroxyl (OH^.^) radicals. As non-thermal plasma generates free radicals, including OH^.^ and $O_{2}^{.-}$, it has a similar decontamination mechanism to hydrogen peroxide. Masks decontaminated using hydrogen peroxide cannot be used immediately due to the risk of hydrogen peroxide residues that are corrosive to skin. Compared to hydrogen peroxide, a decontamination method using non-thermal plasma does not have the risk of residues because it generates hydroxyl radicals by directly ionizing air and the ionized species can be quickly recombined once the system is off.

In this study, we present a new non-thermal plasma generation system to decontaminate FFRs without impacting their performance. The developed plasma system consists of a power processing unit and a surface dielectric barrier discharge (DBD) plasma generator. A hexagonal electrode configuration is used on the surface DBD plasma generator, which is printed on a thin polyamide (Kapton) substrate using conductive inks. SARS-CoV-2 contaminated N95/FFP2 and N99/FFP3 mask samples are treated with non-thermal plasma for various times, and the decontamination efficiency is monitored. The performance of decontaminated mask samples is evaluated by measuring filtration efficiency with and without plasma treatment. The findings of this study could establish a foundation for developing a safe FFR decontamination method for reuse.

## II. MATERIALS AND METHODS

### A. Non-thermal plasma generator

**FIG. 1.**
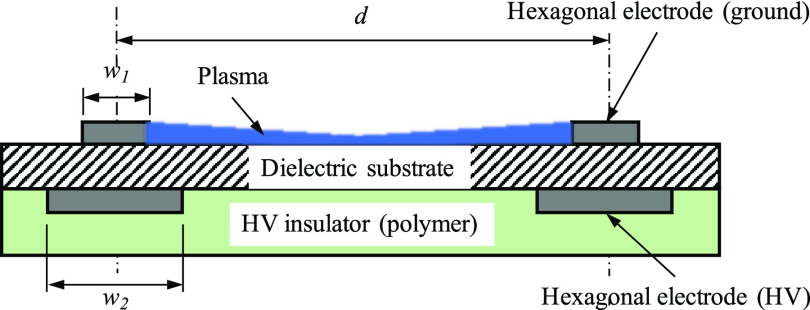
Schematic of the cross section of the surface DBD electrodes used in this study, where *d* is the size of the hexagonal electrode (2 mm), *w*_1_ is the width of the ground electrode (0.3 mm), and *w*_2_ is the width of the high-voltage electrode (0.6 mm).

The non-thermal plasma generator uses a printed electrode with a hexagonal geometry over an area of 38 × 38 mm^2^. As shown in Fig. 1, both high-voltage and ground electrodes are printed on a thin polyamide film (Kapton, 75 *μ*m) as a dielectric barrier with a conductive ink (Conductor2 ink, Voltera). The printed electrodes are cured at 200 ^◦^C for 30 min to ensure the strength and conductivity of the printed electrodes. The printed surface DBD electrodes are used in the head unit of a plasma brush that could help decontaminate FFRs.

**FIG. 2.**
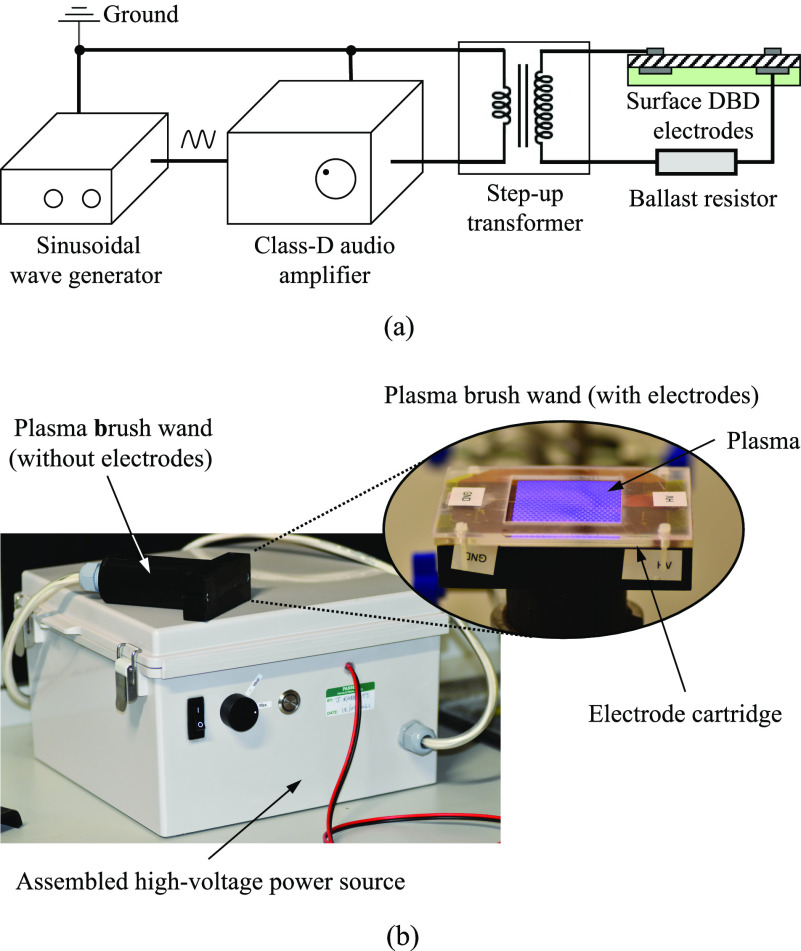
Prototype of the plasma brush decontamination system that consists of a power processing unit, a plasma brush wand, and an electrode cartridge: (a) layout of the plasma brush decontamination system and (b) picture of the assembled plasma brush decontamination system.

Figure 2 shows the prototype of the plasma brush decontamination system using surface DBD. The electrodes generating surface DBD are connected to the high-voltage sinusoidal power source that consists of a sinusoidal wave generator, a class-D audio amplifier, and a high-voltage transformer (CMI-4967, Corona Magnetics). As shown in Fig. 2(a), a ballast resistor is used to protect the system from any excessive currents and to improve the electrical safety of the system. The peak-to-peak voltage of the decontamination system can be adjusted by changing the amplifying output level range of the audio amplifier. The frequency of the amplified high-voltage is determined by the sinusoidal wave generator, which is fixed at 6 kHz in this study. The time evolution of the discharge electrical parameters of the generated plasma is monitored using an oscilloscope coupled with a high-impedance probe (P6015A, Tektronix) for the applied voltage and an inductive probe (Model 6585, Pearson Electronics) for the current. The image of the surface DBD plasma is obtained using an intensified charge-coupled device (ICCD) camera (iStar 340T, Andor). We measure the optical emission spectrum (OES) of the surface DBD plasma using a CCD spectrometer (HR-4000, Ocean optics) that can obtain emission spectra from 200 to 1100 nm wavelengths.

### B. Plasma dose and treatment

Plasma treatment can affect the hydrophobicity of materials due to the formation of high energy surface groups in reactions between surface materials and reactive plasma species.^15^ Reduced hydrophobicity by plasma treatment is typically not stable, and either a partial or complete hydrophobic recovery is usually observed. It is known that the change in hydrophobicity depends on the plasma exposure time and intensity.^15^ In this study, we introduce plasma dose, *D*, to quantify the amount of plasma exposed on a sample mask, which is defined as follows:

$$D=\frac{P\cdot t}{A}\;\;\;\left({J/cm}^{2}\right),$$ (1)

where *P* is the time averaged electrical power of the plasma generator, *t* is the plasma exposure time, and *A* is the plasma treated area. The time averaged electrical power of the plasma generator can be calculated by averaging the instantaneous power as follows:

$$P=\frac{1}{t}\int_{0}^{t}V\left(t^{\prime }\right)I\left(t^{\prime }\right)dt^{\prime },$$ (2)

where *V* and *I* are the voltage and current across the surface DBD plasma generator, respectively.

**FIG. 3.**
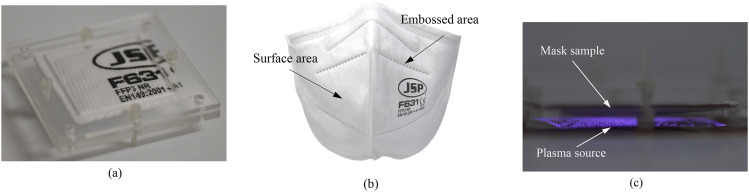
(a) Picture of the prepared mask sample that is cut to a size of 4 × 4 cm^2^. (b) Identification of surface areas of a mask sample. (c) Picture of a mask sample treated with plasma. A plasma generator is placed on the bottom of the mask sample to maintain the distance between the plasma generator and mask sample constant.

Figure 3 shows the used N95/FFP2 (F621, JSP) and N99/FFP3 (F631, JSP) mask samples in this study, which are cut to a size of 4 × 4 cm^2^. A mask sample material of the same size has been used in the filtration and decontamination tests. In this study, both inner and outer sides of masks are tested, which include the surface and embossed areas of mask samples. As the effectiveness of plasma treatment can depend on whether treatments are carried out directly or indirectly,^16^ we have placed mask samples in the field where plasma is generated so that it is in intimate contact with all the reactive species produced.

### C. SARS-CoV-2 treatment and assessment

**FIG. 4.**
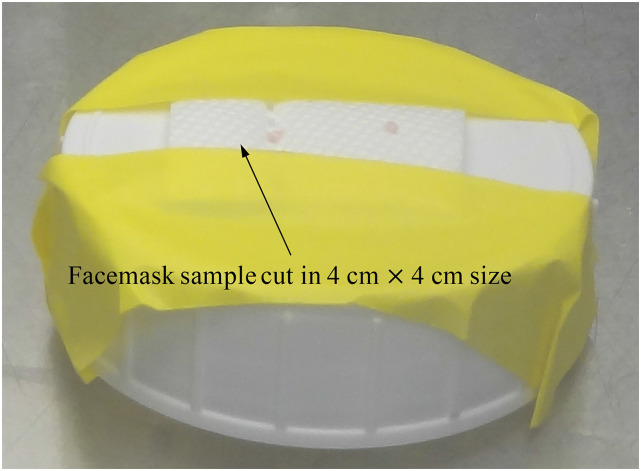
Picture of the test mask sample prepared for decontamination treatment in a Bio-Containment Level 3 cabinet.

We have obtained SARS-CoV-2 (B.1.1.7 strain) from Porton Down, UK, and maintained it in our laboratory. The potential surface contamination of mask samples is modeled using microdroplets (2 *μ*l) of neat virus stock. The microdroplets are pipetted on each sample surface and left to dry for 1 h at room temperature inside a Bio-Containment Level 3 cabinet. As shown in Fig. 4, all tested surfaces are cut to a size of 4 × 4 cm^2^ and taped onto a plastic support for exposure to the plasma source at a comparable distance (1 or 2 mm) using a plastic separator. Spiked surfaces are exposed to plasma for up to 10 min. Recovery control (untreated) and treated spikes are recovered by washing the spiked area using a pipette and resuspending into 1 ml of the infection medium.

We have used SARS-CoV-2-sensible cells to evaluate the virucidal effect of the plasma against SARS-CoV-2. Vero/E6 cells [European Collection of Authenticated Cell Cultures (ECACC), Porton Down, UK] susceptible to SARS-CoV-2 infection are maintained in Dulbecco’s Modified Eagle Medium (Gibco, UK) supplemented with 1% L-glutamine, 10% fetal calf serum, and 1% penicillin and streptomycin. For infectivity tests, Vero/E6 cells are grown to confluency in 12 well plates (Greiner) and washed once with the infection medium (IM: DMEM supplemented with 1% L-glutamine, 2.5% HEPES, and 1% penicillin and streptomycin) prior to incubation (1 h at 37 °C, 5% CO_2_) with samples prepared as described above or standard 1/10 serial dilutions of virus stock in the IM for titration. For each sample, 400 *μ*l is applied per well (in duplicates). After incubation (with intermittent gentle rocking to ensure the homogeneous distribution of virions over cell monolayers), samples are pipetted out and cells are covered with the overlay medium (MEM supplemented with 7.5% sodium bicarbonate, 1% L-glutamine, 2.5% HEPES, 4% FCS, 1% penicillin and streptomycin, and 1.2% Avicel) and maintained at 37 °C, 5% CO_2_ for 3 days. After 3 days, the overlay medium is removed and cells are fixed with 8% (v/v) formalin in phosphate-buffered saline (PBS) for 30 min prior to staining with 0.1% (v/v) crystal violet in 20% methanol for 5–15 min.

**FIG. 5.**
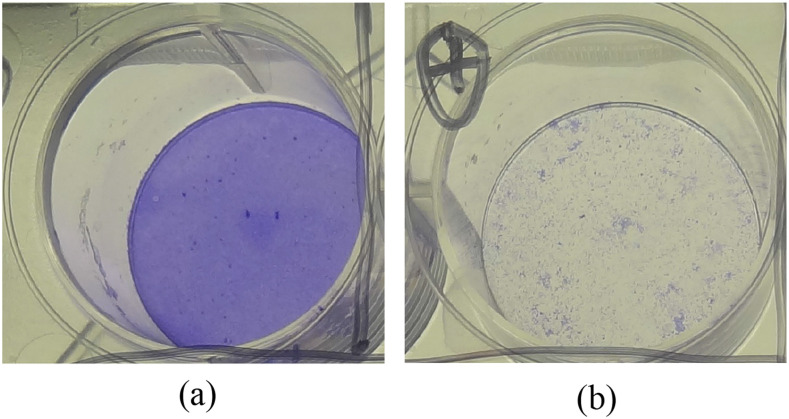
Mask sample preparation and SARS-CoV-2 assessment: (a) negative control (intact monolayer) and (b) recovery control (untreated virus) where all cells are dead.

Figure 5 shows mask samples with recovery and positive controls. We have applied up to 2000 virions in each test sample. The recovery control is washed from the area where the droplet dried. The positive control is the same amount placed directly onto the cells. If there is one virion in the solution applied, it will infect one cell. Thus, a patch hole can be observed in a confluent monolayer. We use cytotoxicity plaques to quantify viral infectivity recovered from surfaces. The obtained results are normalized as a percentage of residual infectivity in applied spikes, which is calculated from the individual titration of the aliquot used on the day, in order to account for slight variations in viral titer between aliquots used for each experiment.

### D. Respirator performance test

**FIG. 6.**
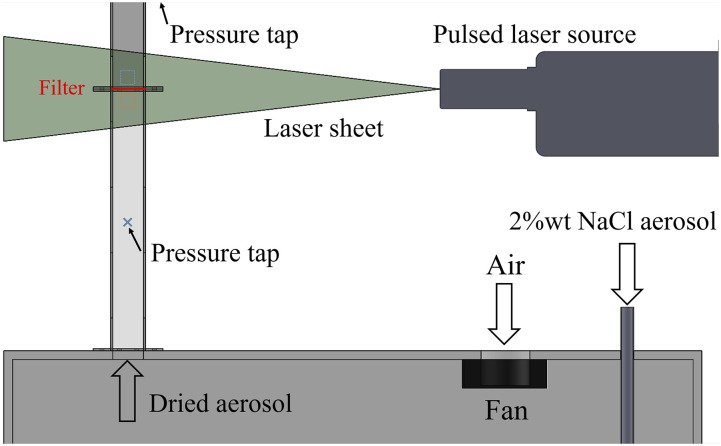
Schematic of the filtration testing setup. A wet NaCl aerosol is fed into the settling chamber via the down tube where it dries. The fan drives the aerosol through the test section and across the filter sample under test, which is clamped in place. The aerosol concentration and flow rate are measured by 2D PIV imaging. The pressure drop is measured across the filter at the pressure taps indicated.

Plasma treated and untreated respirators are tested for filtration efficiency by monitoring the transmission of a sodium chloride (NaCl) aerosol through samples of filter material. Figure 6 illustrates the experimental apparatus used to measure the filtration efficiency. A 2% w/v NaCl solution is atomized using an ATM-230 (Palas, GmbH) Laskin-nozzle aerosol generator, operated with an overpressure of 5 bars. This generates a polydisperse primary aerosol with a median diameter of ∼2 *μ*m, which is allowed to dry within a 0.5 × 0.5 × 0.8 m^3^ settling chamber, yielding a solid NaCl aerosol with an estimated median diameter of 0.5 *μ*m. Aerosol-laden flow is driven through the chamber by the fan into a 35 mm^2^ transparent test section and through a sample of filter material. The pressure drop across the filter material is monitored using an FCO560 micromanometer (Furness Controls) and maintained at 300 ± 30 Pa across tests. The aerosol concentration is measured upstream and downstream of the filter by imaging the flow using a planar particle image velocimetry (PIV) technique. Illumination is provided by a 200 mJ pulsed Nd:YAG laser (Litron Bernoulli 200-15 PIV) whose output is formed into a thin laser sheet of e^−2^ thickness *w* ≈ 0.7 mm. Images are recorded over a region 58 × 38 mm^2^ using a LaVision Imager 16M camera with a 4872 × 3248 pixel sensor and a Sigma 105 mm Macro lens at *f*_#_ = 5.6. The average bulk upstream velocity measured by PIV is 0.39 and 0.34 m/s for the FFP2 and FFP3 samples, respectively.

To obtain the particle concentration, particle images are first pre-processed using a background image subtraction and levelized by the average intensity to enable a uniform identification of particles across images. Particles are identified as local maxima within a calibrated intensity range and counted over equal areas chosen upstream and downstream of the filter. The particle concentration *ρ* = *N*/(*Aw*) is obtained from the average particle count *N* obtained over 1 min (50 images) and the area *A* of the imaged region. As a control, we obtain the transmission *T* = *ρ*_*d*_/*ρ*_*u*_ of the aerosol through the test section with no filter in place. We find *T* = 0.97–1.12 over a range of upstream concentrations (*ρ*_*u*_ = 1.1–3.1 × 10^4^ part/cm^3^).

## III. RESULTS AND DISCUSSION

### A. Plasma characterization

**FIG. 7.**
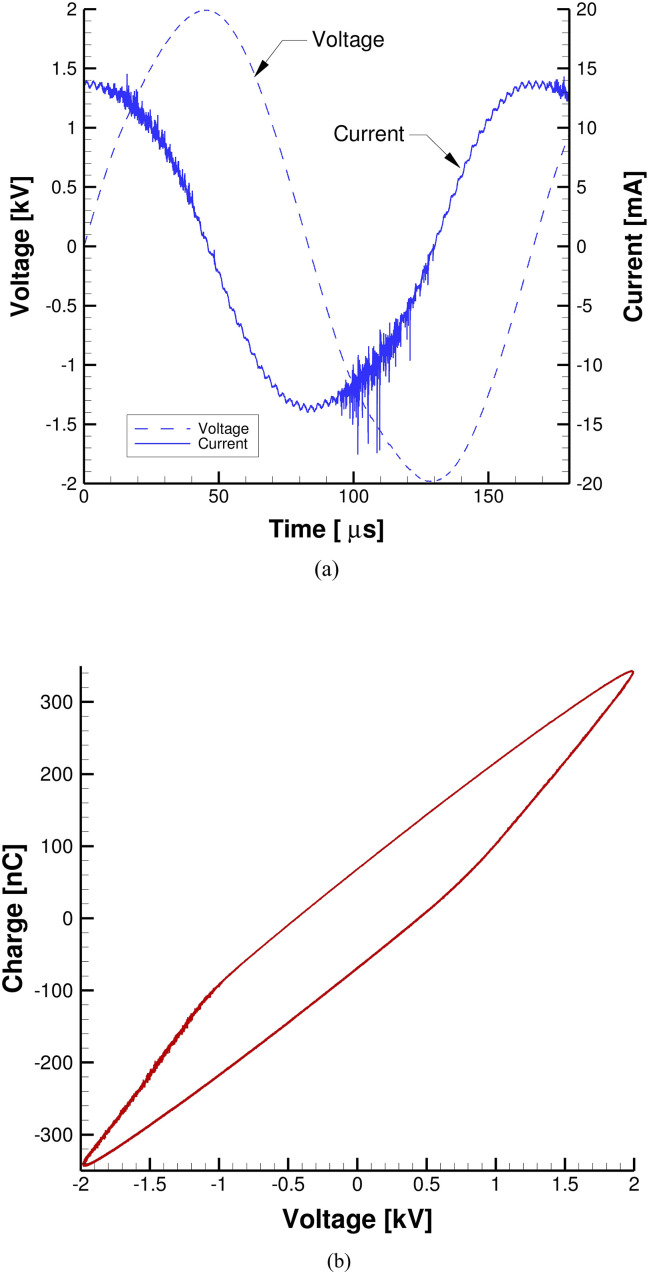
(a) Measured voltage and current and (b) Lissajous figure of the plasma source.

Figure 7(a) shows the measured voltage and current of the plasma decontamination system. The surface DBD plasma source generates uniform plasma across the electrode around 4 kV_*pp*_. The applied voltage is measured directly from the output of the high-voltage transformer using a high-impedance high-voltage probe (P6015A, Tektronix). The charge, *Q*, flowing through the surface DBD plasma source is obtained using an inductive probe (Model 6585, Pearson Electronics). The voltage and charge waveforms are recorded using a digital oscilloscope and used to obtain the Lissajous figure shown in Fig. 7(b) by plotting charge–voltage characteristics. As can be seen, the obtained Lissajous curve is a parallelogram with blunt edges because we use surface DBD, which is different from typical parallelogram-like Lissajous curves derived from volume DBD discharges. We uses the voltage–charge (V–Q) Lissajous method to determine the discharge power in the plasma reactor, thus calculating the plasma dose used in this study. The time averaged power dissipation in the plasma source is estimated from the area of the Lissajous figure and driving frequency using Eq. (2). The dissipated power in the plasma discharge operating is 5.45 W, which is averaged for 5 ms, which is equivalent to ∼26 periods of plasma discharges. Using the obtained plasma discharge power, the plasma dose for the three different treatment times of 5, 10, and 30 min is 113.2, 226.5, and 679.4 J/cm^2^, respectively.

**FIG. 8.**
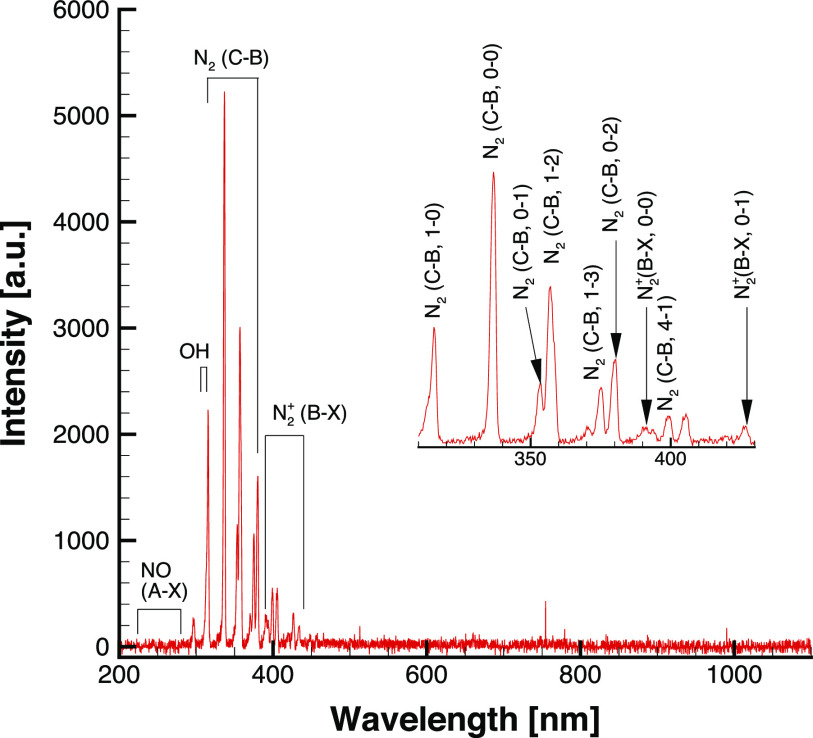
Emission spectra for the DBD plasma generator with 4 kV_*pp*_ at 6 kHz.

The optical emission spectrum (OES) is measured between 200 and 1100 nm from the surface DBD plasma generator to measure the reactive oxygen and nitrogen species, which is shown in Fig. 8. As can be seen, the major emission peaks are formed by the second positive system of nitrogen [N_2_ (C–B)] in the wavelength band from 316 to 380 nm. The optimal emission intensity of the second positive nitrogen is used as a proxy for the overall discharge intensity of the source DBD plasma generator.^17^ The emission peaks of the hydroxyl radicals (OH) at wavelengths 306–312 nm and of nitric oxide (NO) at wavelengths below 300 nm are characterized by lower intensity. The first negative system of nitrogen from 390 to 440 nm is also detected. The measured OES also shows that the ozone is generated as follows:

$$N_{2}+e\to N_{2}^{+}+e+e,$$ (3)

$$N_{2}^{+}+O_{2}\to N_{2}^{+}+O+O,$$ (4)

$$O+O_{2}+M\to O_{3}+M.$$ (5)

These species, including reactive molecular radicals and ozone, will be virucidal agents produced through the electrical breakdown of air. In this study, we permit the contact of plasma and FFR samples. Therefore, both short and long lived reactive species contribute to the decontamination of the FFP2 and FFP3 respirator samples.

### B. Decontamination efficiency

In this study, the results are expressed as mean residual infectivity (%) ± SEM from at least three separate experiments for each condition and are analyzed by analysis of variance (ANOVA) and t-test. A value of *P* ≤ 0.05 is considered significant.

**FIG. 9.**
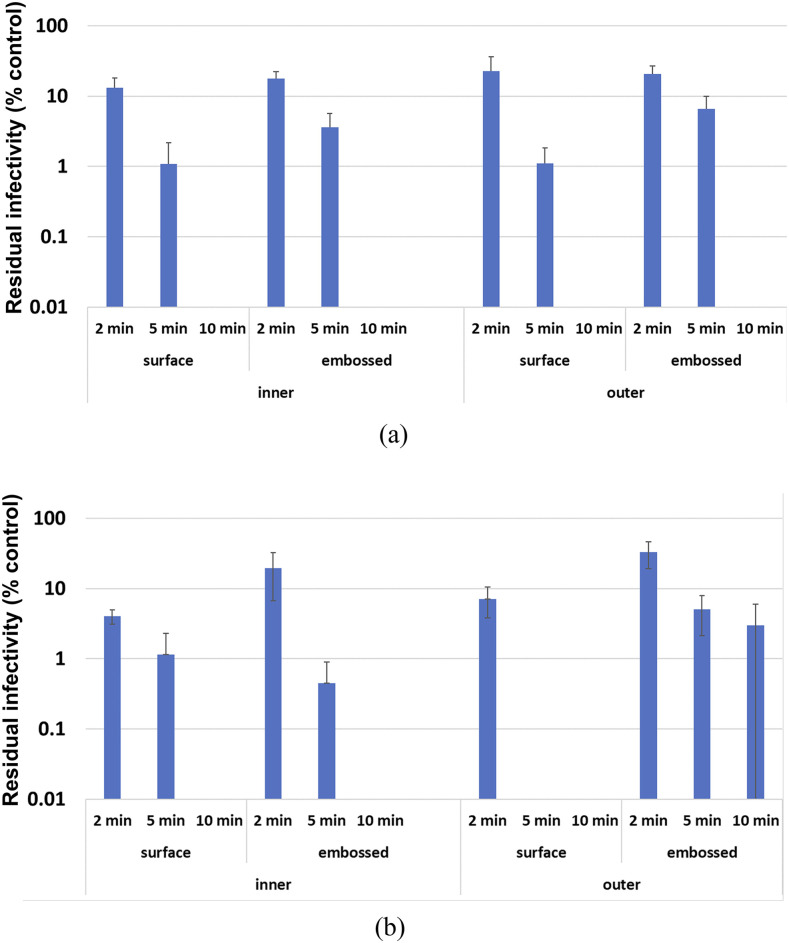
Residual infectivity normalized by the individual titration of the aliquot used on the day: (a) FFP2 mask samples and (b) FFP3 mask samples.

Figure 9 shows the residual infectivity of plasma treated FFP2 and FFP3 mask samples on the logarithmic scale. As can be seen, 2 min plasma treatment has a moderate effect on viral infectivity as some residual cytotoxic plaques are observed from all samples independently of the surface tested. After 5 min of treatment, only a few sample surfaces still harbor infectivity, and after 10 min of treatment, no residual infectivity is recovered from the surfaces tested, except in a single case (embossed area on the outer surface of an FFP3 mask).

A high plasma dose (363 J/cm^2^, 10 min treatment) is required to achieve apparent complete neutralization of viral infectivity from all surfaces tested. The key limitation of this system is to guarantee plasma contact for 10 min on all the surfaces of masks that often present complex geometries. Using our experimental setup, there is little difference in results obtained from plasma sources with either 1 or 2 mm separators, and these are combined for analysis. This is likely due to the flexibility of the plasma generating and treated surfaces (never a perfect plane), creating variations in the gap that are probably within the 1 mm range. Furthermore, the embossed areas on mask samples equate to ∼1 mm depressions. This might explain why treatments of similar duration appeared sufficient in some cases and justifies the use of 10 min treatment to account for such minute variations when treating complex geometries.

### C. Filtration

**FIG. 10.**
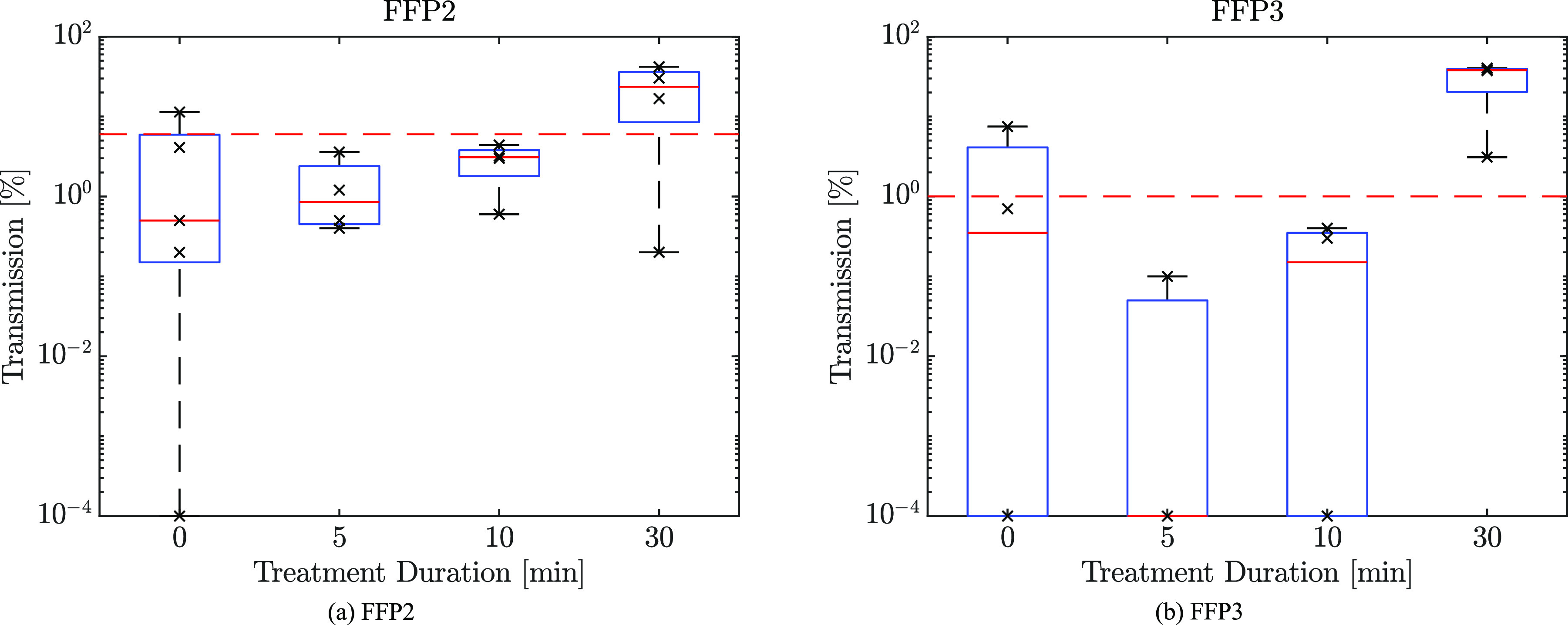
Aerosol transmission through (a) FFP2 and (b) FFP3 respirator materials after 0, 5, 10, and 30 min of treatment, which are equivalent to the plasma doses of 0, 182, 363, and 1090 J/cm^2^, respectively. Markers indicate individual tests. The red dashed line marks 6% and 1% transmission levels. Transmission levels below 10^−4^% are clipped at this level.

The relative filtration performance of FFP2 and FFP3 respirator samples for varying treatment durations is shown in Fig. 10. We observe considerable variability in aerosol transmission across samples. After 5 min of treatment, there is an apparent improvement in filtration performance; increasing the treatment duration causes subsequent degradation of the filtration performance. The obtained results show no significant impact on the filtration efficiencies of both FFP2 and FFP3 respirators up to 10 min of direct plasma exposure, which is equivalent to undertaking two decontamination cycles.

For both untreated FFP2 and FFP3 materials, we find samples that transmit over 6% and 1% of incoming aerosol, respectively. This exceeds the limits outlined in BS-EN 149^1^ for these respirator types. However, there are some differences between our testing procedure and the standard, notably the method of particle concentration measurement, which may account for this discrepancy. We, therefore, cautiously interpret the change in the performance difference in a relative sense. We note that the degradation in filtration efficiency after 30 min treatment is significant, exceeding the standard by an order of magnitude, and is therefore unlikely to meet BS-EN 149 specification.

Reducing the filtration efficiency by long plasma exposure can be related to electrostatic filtration in the FFR. FFRs generally consist of four different layers of filters, which are the outer, filter, support, and inner layers.^18^ The inner and outer layers use hydrophobic non-woven polypropylene to prevent moisture from being absorbed. A modacrylic support layer provides the shape and thickness to the FFR, improving rigidity and comfort. A melt-blown non-woven polypropylene filter layer removes particles through inertial impaction, interception, diffusion, and electrostatic attraction.^18^ The filtration efficiency of a filter layer, therefore, can be expressed as

$$\eta_{s}=1-\left(1-\eta_{R}\right)\left(1-\eta_{I}\right)\left(1-\eta_{D}\right)\left(1-\eta_{E}\right),$$ (6)

where *η*_*R*_, *η*_*I*_, *η*_*D*_, and *η*_*E*_ are the filtration efficiencies by interception, impaction, diffusion, and electrostatic attraction, respectively. Interception, impaction, and diffusion efficiencies are mainly governed by the physical geometries of the filter layer, such as fiber diameter, filter thickness, porosity, and pore diameter.^19–21^

**FIG. 11.**
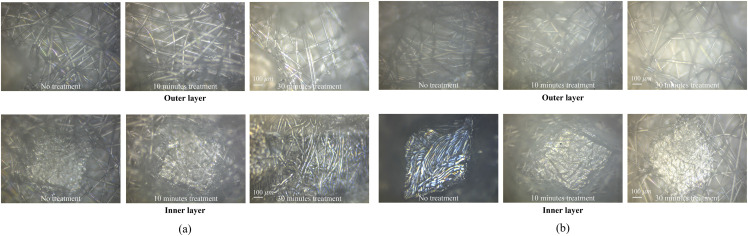
Episcopic differential interference contrast microscopy images of plasma treated mask samples: (a) FFP2 and (b) FFP3.

We examine the filter surfaces by episcopic differential interference contrast (EDIC) microscopy to assess any potential structural damage. As can be seen in Fig. 11, no damage is observed in the microfibre structures after masks are subjected to three successive 10 min-long plasma treatments. As no physical damage has been observed after the plasma decontamination process, the reduced filtration efficiency after 30 min treatment could be caused by reducing the filtration efficiency of electrostatic attraction.

The electrostatic attraction filter efficiencies for both charged and uncharged particles are governed by the electric field strength.^22^ During the plasma decontamination process, respirator samples are exposed to an external AC electric field, about 2 MV/m. When an external AC electric field is applied to a polarized melt-blown non-woven polypropylene filter layer, the polarizability of the filter layer can be affected. The long-term exposure of the AC electric field may lead to a reduction in the filtration efficiency of the filter layer by changing the polarizability of the filter. A further study, therefore, would be needed to quantify the influence of the external AC electric field on the electric field strength of the filter layer.

## IV. CONCLUSION

We have developed a new non-thermal plasma decontamination system that can inactivate SARS-CoV-2 on FRRs using surface DBD. Although most of the FRRs are made for single-use, they can be reused for a limited time if there is no risk of contamination through the deposition of infectious particles on the surface. The developed non-thermal plasma decontamination system has been successfully used to decontaminate N95/FFP2 and N99/FFP3 masks. We have achieved a 4 log reduction for the coronavirus without reducing the filtration efficiency of masks after 5-min plasma exposure, which is equivalent to the plasma dose of 182 J/cm^2^. As the decontamination efficiency of plasma is influenced by both exposure time and plasma intensity, we can use the plasma dose as a parameter to suggest the minimum exposure time depending on the size of PPE and the power of the plasma source, which can develop the appropriate procedure of an actual PPE decontamination process. This study, therefore, is valuable for solving the acute shortages of FFRs in many countries and their environmental and economic burdens against discarding reusable masks.

## Data Availability

The data that support the findings of this study are available from the corresponding author upon reasonable request.
